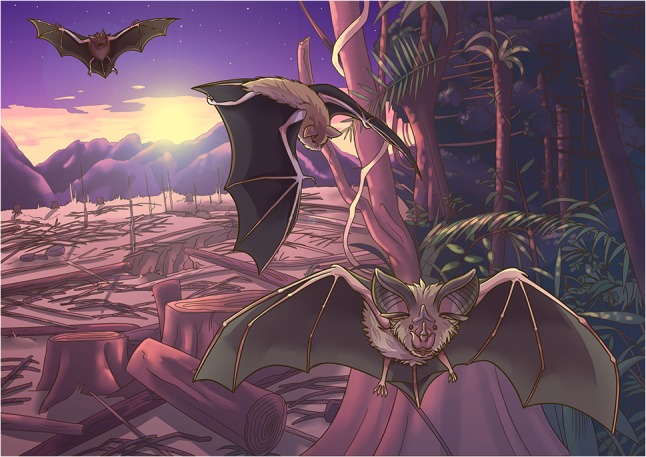# Forest disturbance leaves some bats stressed and under the weather

**DOI:** 10.1093/conphys/cox041

**Published:** 2017-06-27

**Authors:** Christine L. Madliger

**Affiliations:** 1 Department of Biological Sciences, University of Windsor, 401 Sunset Ave., Windsor, Ontario, Canada N9B 3P4

In the face of logging and forest fragmentation, what is a bat to do? Interestingly, the type of roosting site a bat uses may have a big influence on how habitat disturbances affect them. It turns out that bats that roost in caves may have an easier time coping with change.

Traditionally, conservation scientists have focused on population size to measure how disturbances affect species, but this type of monitoring can take years to indicate that there is an issue. This lag time is one reason why some conservation scientists are turning to physiology. Hormones, metabolites and body condition are some of the physiological parameters that can change rapidly when the environment changes. This knowledge can allow conservation practitioners to work proactively to protect wildlife.

Indeed, Seltmann *et al.* (2017) and her team used physiology to help pinpoint the bat species most sensitive to logging in the rainforests of Borneo. They examined eight bat species—some that use caves to roost and some that roost in trees or the cavities of trees (foliage)—to determine if the type of roost site could cause some bats to be more susceptible to the impacts of logging.

To do this, Seltmann and her team captured bats in patches of recovering, fragmented or actively logged forests. They measured three aspects of the bats’ physiology. First, they weighed the bats, because body mass can be a good indicator of overall condition. Bats that have access to sufficient food tend to have a greater body mass. They also took blood samples to measure two other physiological parameters: white blood cell numbers, which indicate disease susceptibility, and neutrophil to lymphocyte ratios, because they rise when stress hormones go up.

Not surprisingly, the researchers found fewer bats in the disturbed sites versus the recovering sites. But forest disturbance only impacted the physiology of some species and barely affected others. Where was the pattern? It seemed that foliage-roosting bats living in disturbed forests weighed less, and some also exhibited weakened immune systems. In contrast, only one species of the cave-roosting bat showed signs of stress.

The effects of a disturbed forest seem to differ based on the type of roost that bats use. But why? The team thinks that foliage-roosting bats may be extremely sensitive to forest disturbance because their roosts are directly affected. When they leave their roosts, stress levels can also elevate and lead to disease and/or starvation. Cave-roosting bats in disturbed forests can retreat to undisturbed caves to roost and may already be used to travelling longer distances to find food in the first place.

Habitat disturbances can have diverse consequences for wildlife, but physiology can help us to understand which species may be affected and in what way. For tropical bats, managers may want to pay close attention to foliage-roosting species that could be more susceptible to disease outbreaks when human activity encroaches on their habitat.

Given that bats provide more than US $1bn worth of pest control globally on corn crops alone, well-informed conservation programs will be crucial for both bats and humans.

Illustration by Erin Walsh; Email: ewalsh.sci@gmail.com

**Figure:**